# Results of haploidentical transplant in patients with donor-specific antibodies: a survey on behalf of the Spanish Group of Hematopoietic Transplant and Cell Therapy

**DOI:** 10.3389/fimmu.2023.1165759

**Published:** 2023-05-26

**Authors:** Rebeca Bailén, Raquel Alenda, Beatriz Herruzo-Delgado, Cynthia Acosta-Fleitas, Ana Vallés, Albert Esquirol, Marta Fonseca, Laura Solán, Irene Sánchez-Vadillo, Guiomar Bautista, Leyre Bento, Oriana López-Godino, Ariadna Pérez-Martínez, Anna Torrent, Joud Zanabili, María Calbacho, Miguel Ángel Moreno, María Jesús Pascual-Cascón, Luisa Guerra-Domínguez, Anabelle Chinea, Irene García-Cadenas, Lucía López-Corral, Francisco Boix-Giner, José Luis López Lorenzo, Karem Humala, Rafael Duarte, Antonia Sampol, Inmaculada Heras, José Luis Vicario, Antonio Balas, Gillen Oarbeascoa, Paula Fernández-Caldas, Javier Anguita, Mi Kwon

**Affiliations:** ^1^ Department of Hematology and Hemotherapy, Hospital General Universitario Gregorio Marañón, Madrid, Spain; ^2^ Translational Oncology Section, Gregorio Marañón Health Research Institute, Madrid, Spain; ^3^ Department of Histocompatibility, Centro de Transfusión de la Comunidad de Madrid, Madrid, Spain; ^4^ Department of Hematology and Hemotherapy, Hospital Universitario Regional de Málaga, Málaga, Spain; ^5^ Department of Hematology and Hemotherapy, Hospital Universitario Doctor Negrín, Gran Canaria, Spain; ^6^ Department of Hematology and Hemotherapy, Hospital Universitario Ramón y Cajal, Madrid, Spain; ^7^ Hematology Department, Hospital de la Santa Creu i Sant Pau, Sant Pau Health Research Institute and Jose Carreras Leukemia Research Institutes, Universitat Autonoma of Barcelona, Barcelona, Spain; ^8^ Department of Hematology and Hemotherapy, Hospital Clínico Universitario de Salamanca, Salamanca, Spain; ^9^ Department of Hematology and Hemotherapy, Hospital Universitario Fundación Jiménez Díaz, Madrid, Spain; ^10^ Department of Hematology and Hemotherapy, Hospital Universitario La Paz, Madrid, Spain; ^11^ Department of Hematology and Hemotherapy, Hospital Universitario Puerta de Hierro Majadahonda, Madrid, Spain; ^12^ Department of Hematology and Hemotherapy, Hospital Universitario Son Espases, Palma de Mallorca, Spain; ^13^ Department of Hematology and Hemotherapy, Hospital Universitario Morales Meseguer, Murcia, Spain; ^14^ Department of Hematology and Hemotherapy, Hospital Clínico Universitario de Valencia, Valencia, Spain; ^15^ Department of Hematology and Hemotherapy, Hospital Germans Trias i Pujol, Barcelona, Spain; ^16^ Department of Hematology and Hemotherapy, Hospital Universitario Central de Asturias, Asturias, Spain; ^17^ Department of Hematology and Hemotherapy, Hospital Universitario 12 de Octubre, Madrid, Spain; ^18^ CIBERONC and Centro de Investigación del Cáncer-Instituto de Biología Molecular y Celular del Cáncer (Universidad de Salamanca - CSIC), Salamanca, Spain; ^19^ Department of Medicine, Universidad Complutense de Madrid, Madrid, Spain

**Keywords:** donor-specific antibodies, graft failure, haplo-HSCT, desensitization strategies, DSA kinetics

## Abstract

**Background:**

Donor-specific antibodies (DSAs) are IgG allo-antibodies against mismatched donor HLA molecules and can cause graft failure (GF) in the setting of haploidentical hematopoietic stem cell transplantation (haplo-HSCT). Our aim was to report the experience of the Spanish Group of Hematopoietic Transplant (GETH-TC) in DSA-positive patients who had undergone haplo-HSCT.

**Methods:**

We conducted a survey of patients who underwent haplo-HSCT in GETH-TC centers between 2012 and 2021. Data were collected on the DSA assay used, monitoring strategy, complement fixation, criteria for desensitization, desensitization strategies and transplant outcomes.

**Results:**

Fifteen centers from the GETH-TC responded to the survey. During the study period, 1,454 patients underwent haplo-HSCT. Seventy of the transplants were performed in 69 DSA-positive patients, all of whom lacked a suitable alternative donor; 61 (88%) patients were female (90% with prior pregnancies). All patients received post-transplant cyclophosphamide-based graft-versus-host disease prophylaxis. Regarding baseline DSA intensity, 46 (67%) patients presented mean fluorescence intensity (MFI) >5,000, including 21 (30%) with MFI >10,000 and three (4%) with MFI >20,000. Six patients did not receive desensitization treatment, four of them with MFI <5,000. Of 63 patients receiving desensitization treatment, 48 (76%) were tested after desensitization therapy, and a reduction in intensity was confirmed in 45 (71%). Three patients (5%) experienced an increase in MFI after desensitization, two of whom experienced primary GF. Cumulative incidence of neutrophil engraftment at day 28 was 74% in a median of 18 days (IQR, 15─20); six patients died before engraftment due to toxicity or infection and eight patients had primary GF despite desensitization in seven of them. After a median follow-up of 30 months, two-year overall and event-free survival were 46.5% and 39%, respectively. The two-year cumulative incidence of relapse was 16% and non-relapse mortality (NRM) was 43%. Infection was the most frequent cause of NRM, followed by endothelial toxicity. Multivariate analysis identified baseline MFI >20,000 as an independent risk factor for survival and an increase in titers after infusion as an independent risk factor for GF.

**Conclusions:**

Haplo-HSCT is feasible in DSA-positive patients, with high rates of engraftment after desensitization guided by DSA intensity. Baseline MFI >20,000 and increased intensity after infusion are risk factors for survival and GF.

## Introduction

Allogeneic hematopoietic stem cell transplantation (allo-HSCT) is the only curative treatment option for several hematological disorders, mainly malignant diseases (1). For those patients who lack an HLA-identical sibling or matched unrelated donor (MUD), the use of alternative donor strategies, including mismatched unrelated donors (MMUDs), umbilical cord blood (UCB) and haploidentical donors, has significantly increased the possibility of allo-HSCT. In fact, the number of haploidentical HSCT (haplo-HSCT) is growing every year (2). However, these transplant modalities have introduced new challenges, including the strategies required in the presence of donor-directed anti-human leukocyte antigen (HLA)-specific allo-antibodies (DSAs).

DSAs are preformed mostly IgG antibodies with specificity against HLA molecules not shared with the donor (3). Their significance in mediating rejection was first described in solid organ transplantation, and has been widely reported to be related to graft failure (GF) in several HSCT settings, including MUD, MMUD, UCB and haplo-HSCT (3–9). Accordingly, screening for DSAs before these procedures is essential (10). DSAs are more frequently reported in the haplo-HSCT setting, due to a higher degree of mismatch and, in the case of female recipients, to the possibility of alloimmunization to offspring antigens following pregnancy (11).

The gold standard technique for the detection of DSAs is based on solid-phase immunoassays (SPI) using the Luminex^®^ platform (12). Although non-quantitative, this approach measures the immunofluorescence intensity of the antibodies, expressed as mean fluorescence intensity (MFI), which has been found to have a clinically significant association with graft failure (GF) (13). It can also be modified to detect complement-fixing DSAs (C1q+ or C3d) (14). In DSA-positive patients, the first option should be to search for donors against whom the recipient does not show DSAs. However, this option is not always available, and in those cases where alternative suitable donors cannot be found, desensitization strategies should be considered if therapeutic options other than transplantation are not possible. In recent years, different groups have reported multimodal desensitization strategies based on those developed for solid organ transplantation (10, 11, 14–16), including a combination of strategies that remove (therapeutic plasma exchange [TPE]) or neutralize (incompatible platelet or buffy coat transfusion) preformed antibodies, reduce their production (rituximab [RTX], bortezomib or immunosuppressive therapies), or inhibit the complement cascade (IV immunoglobulins [IVIG]). The use of these techniques has reduced the incidence of GF to less than 10% (17). However, no study has compared the different combination strategies, and the procedure depends on center policies and experience. Only one set of consensus guidelines has been published thus far by the European Society for Blood and Marrow Transplantation (EBMT), in 2018 (11). More recently, the MD Anderson Cancer Center (MDACC) and City of Hope group has reported the outcomes of an homogeneously-desensitized cohort of 37 DSA-positive patients who underwent haplo-HSCT previously treated with TPE, IVIG, RTX and buffy coat (18), comparing them with those of a haplo-HSCT cohort without DSAs.

Recently, the Madrid Group of Hematopoietic Transplant (GMTH) reported the effect of desensitization treatments guided by DSA intensity kinetics in a cohort of 19 patients (19). In this regard, the aim of the present study within the Spanish Group of Hematopoietic Transplant and Cell Therapy (GETH-TC) was to confirm these results in a large cohort of patients across Spain.

## Patients and methods

### Patients and variables

A survey was sent to all centers belonging to the GETH-TC to identify patients with DSAs who underwent haploidentical donor transplantation. Information was collected on DSA screening, monitoring strategy and desensitization strategy used, together with pre-transplant characteristics and outcomes. The study period included patients transplanted between January 2012 and December 2021. Disease and transplant characteristics were collected from the electronic records of each center. The study was conducted according to the Declaration of Helsinki and approved by the ethics committee of the Hospital General Universitario Gregorio Marañón. All patients signed informed consent.

### HLA typing and DSA identification

HLA typing was performed at high/intermediate resolution using DNA-based techniques (20) in two different samples. Haploidentical donors were all related donors sharing a single HLA haplotype; patients were studied 10 loci (HLA-A, -B, -C, -DRB1 and -DQB1). Patient sera was collected from clotted samples to perform Luminex^®^ SPI IgG single antigen tests (Lifecodes, Immucor, USA or One Lambda, ThermoFisher Scientific Brand, USA), covering HLA-A, -B, -C, -DRB1, -DRB3, -DRB4, -DRB5, -DQA1, -DQB1, -DPA1 and -DPB1 antigens (12). Units were expressed as raw MFI and normalized MFI respectively. Some patients were also tested for complement-fixing antibodies using C1q or C3d techniques. For data analysis, IgG MFI values between 1,000 and 5,000 were considered low; between 5,000 and 10,000 intermediate; and >10,000 high, as reported by the EBMT consensus (11).

### DSA monitoring and desensitization strategy

The survey included policies followed for both DSA monitoring and the desensitization strategy used. DSA screening was performed once the mismatched HSCT was indicated. Some centers also analyzed additional samples prior to desensitization, prior to infusion and/or post infusion according to their policies and experience. Desensitization strategies used also varied according to center policies and experience, immunofluorescence intensity, type of antibodies (anti-HLA class I and/or anti-HLA class II) and complement fixation, if available. Treatments included RTX, IVIG, TPE, incompatible platelet transfusions, buffy coat transfusion, immunosuppressive agents (mycophenolate mofetil [MMF], tacrolimus and steroids) and bortezomib. The combination of treatment used and decision on whether to treat or not depended on the center policy and was analyzed separately.

### Pre- and post-transplant evaluation

Patients were stratified according to the disease risk index (21). Pre-transplant comorbidities were evaluated using the HSCT comorbidity index (HCT-CI) (22). Acute graft-versus-host disease (aGVHD) was scored according to MAGIC criteria (23), and chronic GVHD (cGVHD) was scored according to the NIH Consensus Development Project (24). Myeloid engraftment was defined as an absolute neutrophil count (ANC) of 0.5×10^9^/L or greater for three consecutive days. Platelet engraftment was defined as a platelet count of 20×10^9^/L or higher without transfusion support for three consecutive days. Patients who survived more than 28 days and failed to achieve myeloid engraftment were evaluated on a case-by-case basis to discard possible graft failure. Diagnosis of disease recurrence was based on clinical and pathological criteria.

### Study variables

The primary endpoints were rates of myeloid and platelet engraftment. Secondary endpoints included occurrence of aGVHD, cGVHD, endothelial toxicity, relapse, non-relapse mortality (NRM) or death from any cause. Relapse, toxic death and second transplant due to GF were considered events for event-free survival (EFS). Analysis performed for overall survival (OS), EFS, relapse, NRM, GF and endothelial toxicity included the HSCT period (2013─2017 vs. 2018─2021), patient and donor sex, patient age, number of pregnancies (0, 1─2, >2), prior HSCT, HCT-CI (0─2 vs. >2), cytomegalovirus (CMV) sero-status (iso vs. no, donor negative/recipient positive vs. other), ABO incompatibility (none vs. minor vs. major), intensity of conditioning regimen (myeloablative vs. reduced-intensity), stem cell source (peripheral blood stem cells [PBSC] vs. bone marrow [BM]), CMV reactivation before day 100, DSA class (I vs. II vs. both), baseline intensity of DSAs (<5,000 vs. 5,000─9,999 vs. 10,000─19,999 vs. >20,000 MFI), baseline complement fixation, increase after infusion and use of a desensitization strategy (none, RTX-based, non RTX-based). Last update of the cohort was performed in December 2021.

### Statistical analysis

Qualitative variables were expressed as frequency and percentage. Quantitative variables were expressed as median and either interquartile range (IQR) (25th and 75th percentiles) or range. The χ2 test was used to identify correlations between qualitative variables, and the non-parametric Kruskal-Wallis test was used for quantitative variables. Variables that were significantly correlated in the univariate analysis were evaluated using a forward stepwise selection method with a *p*-in value of <0.05 and a *p*-out of <0.1. The criterion for inclusion in multivariate Cox regression analysis was a *p*-value of <0.1. Estimates of EFS and OS were calculated using the Kaplan-Meier method. Cumulative incidence curves and competing risk regression were performed as alternatives to Cox regression for survival data in the presence of competing risks. In our case, the only competing event for engraftment was toxic death prior to day 28. NRM and relapse were considered competing events for each other, in addition to a second transplant for both of them. Univariate and multivariate analyses were performed with SPSS (IBM, SPSS Statistics for Windows, Version 21.0. Armonk, NY, USA) and Stata 17 for Windows. Cumulative incidence was calculated with R Studio version 1.0.2.

## Results

### Patient and transplant characteristics

Between January 2012 and December 2021, 1,454 patients underwent haplo-HSCT in 15 centers in Spain. Among those, a total of 69 patients with DSAs underwent haplo-HSCT from a donor against whom DSAs were present in the recipient, with a total of 70 HSCTs performed between November 2013 and July 2021.

The characteristics of the 69 patients and transplants are shown in **Table 1**. Sixty-one patients (88%) were female, and 90% of them had prior pregnancies. All patients had received multiple transfusions prior to transplant. The most frequent diagnoses were acute myeloid leukemia and high-risk myelodysplastic syndrome. Patients with prior transplant relapsed after the first transplant and had an urgent indication for a second procedure. All patients lacked an alternative donor against whom no DSAs were present; haplo donors were selected according to MFI and donor characteristics, prioritizing younger donors. Most patients received PBSCs.

**Table 1:** Patient and transplant characteristics.

	Patients (n=69)
Sex (female, %)	61 (88)
Age (median, IQR)	55 (46-61)
Diagnosis (*n*, %): • AML/MDS • ALL • Non-Hodgkin lymphoma • Hodgkin lymphoma • Plasma cell leukemia • Myelofibrosis • Severe aplastic anemia	49 (71.5) 8 (12) 5 (7) 4 (6) 1 (1.5) 1 (1.5) 1 (1.5)
Disease risk index (*n*, %) • Low • Intermediate • High/Very High • Not-applicable	4 (6) 35 (50.5) 29 (42) 1 (1.5)
HCT-CI score (*n*, %) • 0-2 • ≥3	36 (52) 33 (48)
Sensitization events (*n*, %) • Polytransfusion only • 1-2 pregnancies + polytransfusion • 3-4 pregnancies + polytransfusion	14 (20) 39 (57) 16 (23)
Prior HSCT (*n*, %) • Autologous • Allogeneic • MSD • MUD • Haploidentical	4 (6) 4 (6) 1 2 1
Donor (*n*, %) • Sibling • Child • Parent • 2nd degree relative	27 (40) 37 (53) 3 (4) 2 (3)
Stem cell source PB (*n*, %)	62 (90)
Graft counts • CD34+ cells (x10^6^/kg) (median, range) (PB) • TNC (x10^8^/kg) (median, range) (BM)	6.6 (2.5─15) 2.7 (1.5─6.7)
Conditioning regimen intensity (*n*, %) • Myeloablative • Reduced intensity • Sequential regimen	32 (46) 35 (51) 2 (3)
CMV serostatus (*n*, %) • Donor and recipient positive • Donor and recipient negative • Donor negative, recipient positive • Donor positive, recipient negative	49 (71) 4 (6) 15 (22) 1 (1)
ABO incompatibility (*n*, %) • None • Major • Minor • Bidirectional	53 (77) 6 (9) 9 (13) 1 (1)

ALL, acute lymphoid leukemia; AML, acute myeloid leukemia; BM, bone marrow; CMV, cytomegalovirus; HSCT, hematopoietic stem cell transplant; HCT-CI, Hematopoietic Cell Transplantation-Comorbidity Index; IQR, interquartile range; MDS, myelodysplastic syndrome; MSD, matched sibling donor; MUD, matched unrelated donor; PB, peripheral blood; TNC, total nucleated cell count. One patient with DSAs underwent two transplants; the second procedure is not described.

Myeloablative conditioning regimens were used in 32 (46%) patients, including FluBux regimen in 19 (fludarabine [Flu] 40 mg/m^2^/day on days -6 to -3 plus 3 or 4 days of IV busulfan [Bux] 3.2 mg/kg/day on days -6 to -3); ten received the TBF regimen (Flu, Bux, thiotepa [TT] 5 mg/kg/day (2-3 days)) and three received total body irradiation [TBI]-based regimens with either Flu, VP-16 or cyclophosphamide (Cy). Thirty-five patients (51%) received a reduced intensity conditioning (RIC) regimen: 17 with a modified Baltimore protocol consisting of Flu 30 mg/m^2^/day days -6 to -2, Cy 14.5 mg/kg/day on days -6 and -5 and Bux 3.2 mg/kg/day either one or two days on days -3 and -2 (one patient received TBI instead of Bux); 17 patients received a RIC TBF regimen and one patient was conditioned with clofarabine and melphalan (CloMel). Two patients received a sequential transplant due to active disease with Clo and Ara-C followed by RIC conditioning.

All patients received GVHD prophylaxis with high-dose post-transplant Cy 50 mg/kg/day on days +3 and +4 together with MMF 10 mg/kg/day (except for five patients) and either tacrolimus (46 patients, 67%) or cyclosporine A (23 patients, 33%) since day +5. Three patients (4%) also received ATG.

### DSA characteristics, kinetics and management

Characteristics and kinetics of DSAs are shown in **Table 2**. At baseline, 46 patients (67%) presented intensity >5,000 MFI including 21 (30%) with intensity >10,000, of whom three (4%) presented intensity >20,000 MFI. Complement fixation techniques were performed in 20 patients (29%) and showed fixation in 14. A total of 63 patients (91%) received some desensitization treatment. Reasons for not performing desensitization treatment included low intensity of DSAs (4 patients with intensity <5,000 and 1 with a limit intensity of 6,800) and lack of specific guidelines in one patient who was transplanted in 2014. In the remaining 63 patients, desensitization treatment was used, including 19 patients with MFI <5,000.

**Table 2:** Donor specific antibodies: characteristics, kinetics and management.

	Patients (n=69)
Baseline DSA characteristics (*n*, %) • DSA anti-MHC class I only • *Intensity >5,000 MFI* • DSA anti-MHC class II only • *Intensity >5,000 MFI* • DSA anti-MHC class I and II • *Intensity >5,000 MFI*	33 (48) *24* 19 (27) *7* 17 (25) *15*
Baseline DSA intensity (*n*, %) • >5,000 MFI • >10,000 MFI • >20,000 MFI	46 (67) 21 (30) 3 (4)
Complement fixation techniques available (*n*, %) • *Positive C1q/C3d fixation*	20 (29) *14*
Patients receiving desensitization (*n*, %) • Rituximab • IVIG • TPE • Incompatible platelet transfusion • MMF • Tacrolimus • Buffy coat • Bortezomib • Steroids	63 (91) 53 (84) 42 (67) 33 (52) 26 (41) 26 (41) 13 (21) 12 (19) 2 (3) 1 (2)
Monitoring performed after desensitization (*n*, %) • Patients tested after desensitization • Patients with reduction in intensity • Median reduction in intensity (median %, range)* • Persistent DSA >5,000 MFI at infusion • Persistent DSA >10,000 MFI at infusion • Increase after infusion	(% of 63 desensitized patients) 48 (76) 45 (71) *100 (12*─*100)* 11 (17) 3 (5) 3 (5)

DSA, donor-specific antibodies; IVIG, intravenous immunoglobulin; MFI, mean fluorescence intensity; MHC, major histocompatibility complex; MMF, mycophenolate mofetil; TPE, therapeutic plasma exchange.

Combinations of desensitization treatment used are described in **Supplementary Figure 1**. Treatments used included weekly RTX 375 mg/m^2^ in 53 patients (84% of those treated) (median 2 doses, range 1─6); IVIG 0.4 mg/kg/day in 42 (67%) (median 5 days, range 1─20); TPE in 33 (52%) (median 3 sessions, range 1─10); incompatible platelet transfusion only if class I DSAs were present on days -1 and/or 0 in 26 (41%, median 4.5 pools, range 1─10); MMF 5-10 mg/kg/bid in 26 (41%) (median 14 days, range 7─35, starting 2 to 4 weeks before the infusion date and until day -2); buffy coat only if class II DSAs were present on day -1 in 12 (19%); tacrolimus 0.06 mg/kg/day in 13 (21%); bortezomib in 2 (3%); and dexamethasone in 1 (2%).

Most treatment combinations were personalized, and strategies varied between centers and depending on the period and DSA characteristics. Regarding patients with intensity <5,000 MFI (23 patients, 33%), four (17%) did not receive desensitization, five (22%) received only one treatment strategy without combination and 14 (61%) received 2-3 treatments combined. Among those with intensity >5,000 MFI (46, 67%), 44 (95%) received desensitization, with at least two treatments in 40 (87%).

After or during desensitization therapy, DSAs were monitored in 48 patients (76% of those desensitized) prior to infusion on days -1 or 0. Forty-five (71%) showed a reduction in intensity, with a median reduction in intensity of 100% (range 12─100%, mean 73%). Eleven (17%) patients showed persistent intensity of >5,000 MFI at infusion and three (5%) of >10,000. Post-infusion monitoring was performed in 14 patients (22% of those desensitized). Three patients (5%) showed an increase in immunofluorescence after infusion since day 0, two of whom developed primary GF requiring a second salvage HSCT.

### Engraftment and graft failure

**Figure 1:**
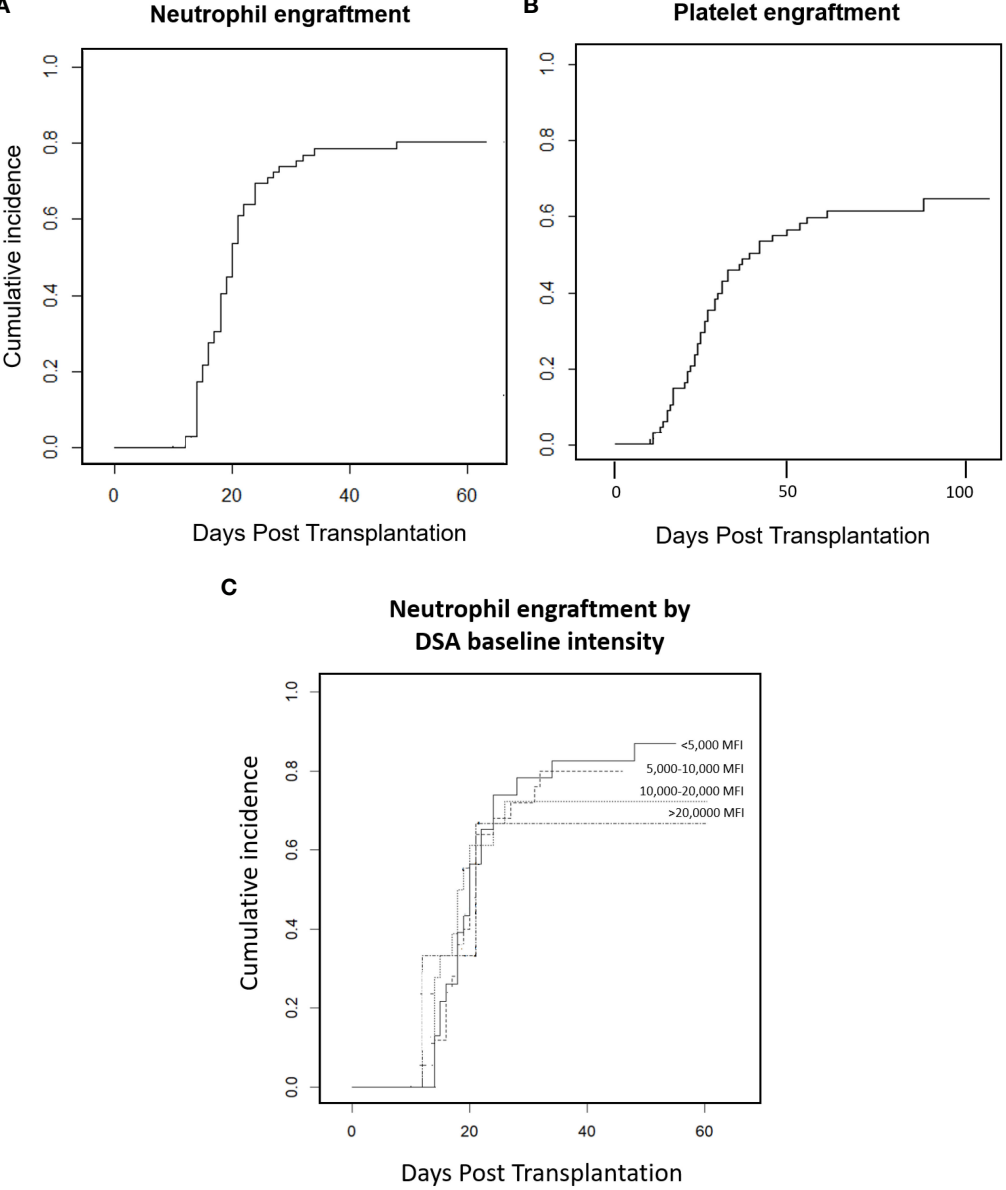
Engraftment. **(A)** Neutrophil engraftment. **(B)** Platelet engraftment. **(C)** Neutrophil engraftment by DSA baseline intensity.

Cumulative incidence of neutrophil recovery at day 28 was 74% and at day 60 was 80% (**Figure 1**). Fifty-five (79%) patients achieved myeloid engraftment in a median of 18 days (range 12─46; IQR 15─20 days). Four of these patients engrafted later than day 28; three of them had shown severe complications that explained the delay in engraftment, including infection and endothelial toxicity. Six patients (9%) died without engraftment prior to day 28 due to toxicity. The cumulative incidence of platelet engraftment at day 28 was 35% and 65% at day 100. Forty-eight (69%) patients achieved platelet engraftment in a median of 31 days (range 11-292, IQR 21-54). Two patients developed secondary GF in the context of CMV infection and 16 (23%) met criteria of poor graft function after engraftment.

**Figure 2:**
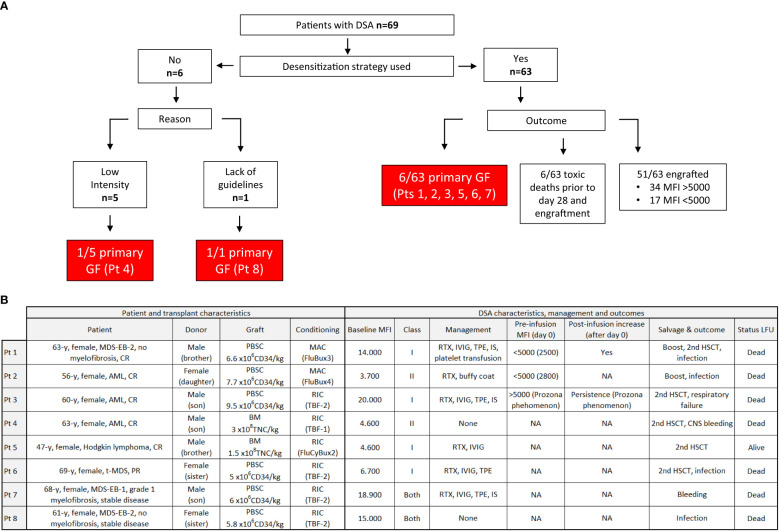
Primary graft failure. Flow of patients according to baseline MFI and desensitization therapy. MFI, mean fluorescence intensity; DSA, Donor specific antibodies; GF, Graft failure; Pt, patient; MDS-EB, myelodysplastic syndrome with excess blasts; CR, complete remission; AML, acute myeloid leukemia; T-MDS, therapy-related MDS; TNC, total nucleated count; MAC, myeloablative conditioning; RIC, reduced intensity conditioning; RTX, rituximab; IVIG, intravenous immunoglobulins; TPE, therapeutic plasma exchange; IS, immunosuppressors; HSCT, hematopoietic stem cell transplantation; CNS, central nervous system; LFU, last follow-up.

Eight patients (12%) developed primary GF, six of them despite desensitization treatment. Regarding potential causes of primary GF in those patients who presented this complication (**Figure 2**), we identified absence of desensitization therapy in two out of eight (25%), of whom one showed high intensity DSAs and was not desensitized and the other showed limit intensity of 6,800; neither of these patients was tested for complement fixation. Among the other six patients, two (25%) showed MFI <5,000 (complement was not tested), one with DSAs against class I and the other against class II. One of them received a bone marrow graft with a total nucleated count under 2x10^8^/kg; none of them engrafted at day 28 although both were rescued, one after infusion of a CD34+ selected boost and the other with a second haplo donor against whom no DSAs were present at that time (alive at last follow-up). Regarding the four patients (50%) who had MFI >5,000 and primary GF despite desensitization, complement was tested in one (which showed fixation), three presented anti-class I DSAs, and one both anti-class I and II. Despite intensive desensitization with at least three treatments including RTX in all of them, one patient showed an increase in intensity after the initial decrease and values <5,000 at infusion, while one patient showed a prozone phenomenon, with saturation of the technique at infusion that prevented detection of very high intensity DSAs at infusion. All four patients died, one prior to salvage therapy because of bleeding and three after a second salvage HSCT because of infection and respiratory failure, one of them after initial engraftment.

In the multivariate analysis performed for GF, increased intensity after infusion was identified as an independent risk factor (adjusted odds ratio (aOR) 24.6 (95% CI, 1.6-365), *p* = 0.020) (**Table 3**).

**Table 3:** Results of univariate and multivariate analyses.

Variables	Overall survival				Graft failure			
	sHR (95%CI)	*p*-value	asHR (95%CI)	*p*-value	OR (95%CI)	*p*-value	aOR (95%CI)	*p*-value
Period (2018─2021)	1.09 (0.54─2.24)	0.801	–	**-**	1.46 (0.26─8.07)	0.665	–	**-**
Sex (male)	0.82 (0.29─2.31)	0.709	–	–	0.97 (0.10─9.37)	0.986	–	**-**
Prior HSCT	0.83 (0.27─2.59)	0.749	–	–	**-**	**-**	–	**-**
HCT-CI ≥3	1.10 (0.60─2.05)	0.758	–	–	1.5 (0.34─6.71)	0.596	–	–
Donor sex (male)	0.67 (0.36─1.23)	0.193	–	–	1.20 (0.26─5.59)	0.818	–	–
Iso-CMV sero-status	0.75 (0.35─1.61)	0.461	–	**-**	0.75 (0.12─4.29)	0.747	–	–
Source (bone marrow)	1.12 (0.43─2.94)	0.820	–	**-**	4.25 (0.63─28.7)	0.138	–	–
Reduced intensity conditioning	1.87 (1.02─3.44)	**0.044**	1.83 (0.99─3.68)	0.052	3.6 (0.66─19.7)	0.140	–	–
Rituximab-based desensitization treatment	1.03 (0.28─3.75)	0.956			0.63 (0.21─1.84)	0.396		
Pregnancies (>2)	0.97 (0.42─2.22)	0.940	**-**	**-**	1.00 (0.15─6.42)	1.000	–	–
Major ABO incompatibility	0.73 (0.20─2.73)	0.649	**-**	**-**	1 (0.32─12.1)	0.452	–	–
CMV reactivation	0.92 (0.45─1.92)	0.839	**-**	**-**	0.78 (0.07─8.43)	0.842	–	–
DSA class (I vs. II vs. both)	0.89 (0.47─1.69) 0.71 (0.29─1.74)	0.732 0.464	**-**	**-**	0.78 (0.12─44.84) 0.89 (0.14─5.58)	0.791 0.904	–	–
DSA intensity >20,000 MFI at baseline	2.92 (1.51─5.63)	**0.001**	1.72 (1.09─2.94)	**0.046**	3.33 (0.22─50.1)	0.384	–	–
Complement-fixation at baseline	2.53 (0.44─14.6)	0.300	**-**	**-**	0.57 (0.06─5.30)	0.622	–	–
DSA intensity >5,000 MFI at infusion	1.13 (0.50─2.57)	0.765	**-**	**-**	2.62 (0.49─13.9)	0.257	–	–
Intensity increase after infusion	1.32 (0.46─3.82)	0.599	**-**	**-**	24.67 (1.67─365)	**0.020**	24.67 (1.67─365)	**0.020**

Values are expressed as absolute numbers and percentages, and hazard/odds ratio and 95% confidence interval. Significant differences are shown in bold. Cox regression analysis was adjusted by the most significant clinical characteristics using a forward stepwise method (see Statistical Analysis section). Criteria for inclusion of covariates in the multivariate analysis were p-value < 0.1. 95%CI, 95% confidence interval; sHR, pseudo hazard ratio; asHR, adjusted pseudo hazard ratio; OR, odds ratio; aOR, adjusted odds ratio; p-value, level of significance. Significant values are marked in bold.

### Survival, relapse and non-relapse mortality

**Figure 3:**
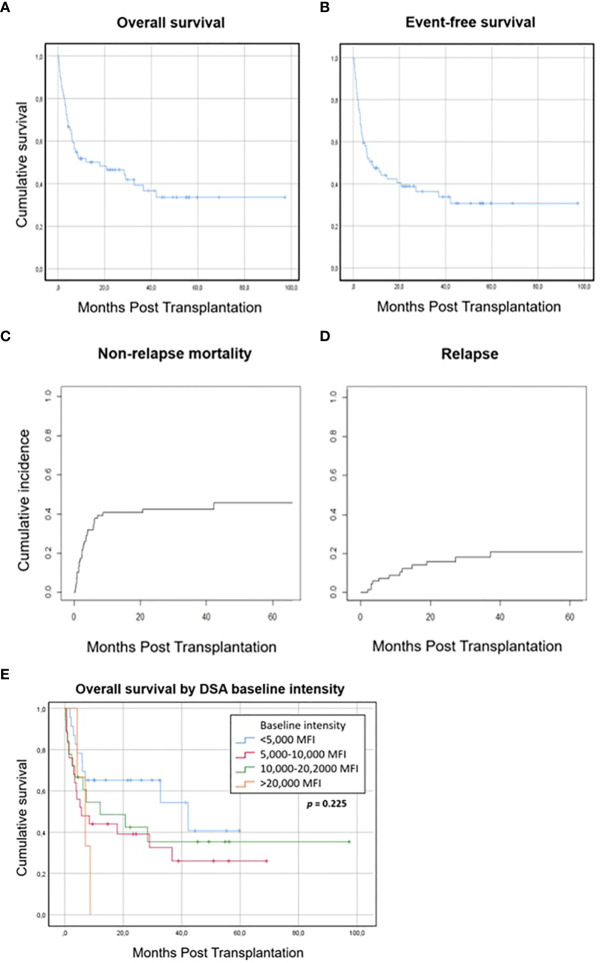
Survival, relapse and non-relapse mortality. **(A)** Overall survival. **(B)** Event-free survival. **(C)** Non-relapse mortality. **(D)** Relapse. **(E)** Overall survival by DSA baseline intensity.

After a median follow-up of 30 months (range 4.5─97 months), the two-year overall survival (OS) and event-free survival (EFS) were 46.5% (and 39%respectively (**Figure 3**). In the multivariate analysis, DSA intensity >20,000 MFI was identified as an independent risk factor for OS (adjusted pseudo-hazard ratio (asHR) 1.72 (95% CI, 1.1─2.9), *p* = 0.046); use of a RIC regimen was the only independent risk factor identified for EFS (asHR 2.11 (95% CI, 1.2─3.8), *p* = 0.013). Two-year survival rates were 65%, 39%, 42% and 0% for patients with baseline DSA intensity of <5,000 MFI, 5,000─10,000 MFI, 10,000─20,000 MFI and >20,000 MFI, respectively.

Cumulative incidence of relapse (CIR) at 2 years was 16% and cumulative incidence of NRM at 2 years was 42.6%. The multivariate analysis identified both use of a RIC regimen (asHR 2.26 (95% CI, 1.1─4.8), *p* = 0.032) and female donor (asHR 2.23 (95% CI, 1─5), *p* = 0.048) as risk factors for relapse, while no independent risk factors were identified for NRM.

A total of 41 (59%) patients died during the study period (**Supplementary Table 1**). Seven of the eight patients who experienced primary GF died (12%): four underwent a second haplo-HSCT, one of them with DSAs against the donor; two died due to complications of the second procedure; and one engrafted and was alive at last follow up. Ten patients (17%) died due to relapse of the underlying disease. Nine patients (22%) died as a result of infection not related to GVHD (7 bacterial, 2 viral). Nine patients (22%) died because of endothelial and CNS complications, including four patients with sinusoidal obstruction syndrome (SOS), two with hemorrhage (one CNS hemorrhage in the context of platelet transfusion refractoriness and one because of diffuse alveolar hemorrhage), one with transplant-associated microangiopathy, one with capillary leak syndrome and one because of CNS demyelinating disease. Finally, six patients (15%) died due to GVHD, three because of refractoriness to treatment and three due to infection under intensive immunosuppressive treatment.

### Toxicity and GVHD

Bacterial infections occurred before day 30 in 23 patients (33%); 18 of them were bloodstream infections. Two patients (3%) were diagnosed with breakthrough invasive fungal infection and 24 (35%) presented early viral reactivations including CMV and BK virus-related cystitis. Forty-five patients (65%) experienced at least one CMV reactivation in the first 6 months after HSCT.

Endothelial complications were diagnosed in 14 patients (20%), including ten patients (15%) who met criteria for SOS (one mild case related to prior inotuzumab ozogamicin treatment and nine moderate to very severe cases accounting for four related deaths) and four cases (6%) of TA-TMA (one patient died due to TMA and two patients died with a concomitant diagnosis of SOS and CMV disease). Finally, one patient (1.5%) presented severe capillary leak syndrome, developing toxic epidermal necrolysis and died. We did not identify any independent risk factor for the development of endothelial toxicity in the multivariate analysis performed.

Four patients (6%) developed neurological complications, including two patients who developed posterior cord syndrome during CMV reactivations, one patient who presented demyelinating disease of the CNS and one patient who developed Guillain-Barré-like demyelinating polyneuropathy of unknown origin, achieving a complete response after IVIG treatment.

The cumulative incidence of grade II-IV aGVHD at day 180 was 29% and grade III-IV aGVHD was 13% at day 180. Cumulative incidence of chronic GVHD at 10 months was 25%, with 15% cumulative incidence of moderate-severe cGVHD (**Supplementary Figure 2**).

## Discussion

DSAs play a major role as a risk factor for GF in mismatched HSCT, which presents as one of the barriers for haplo-HSCT (5, 14, 25). Until the publication of the EBMT guidelines in 2018 (11), there was a lack of recommendations for DSA management and monitoring that have led to heterogeneous management in this setting. Thus, centers have based their policies on their own experience, the desensitization approaches described by groups with more experience, and the accessibility of treatments and monitoring techniques. Verifying the results of these policies is both pertinent and useful to improve the strategies in this poorly described population, and to identify risk factors for GF and survival. In an attempt to report the current practice and outcomes of haplo-HSCT in DSA-positive recipients in Madrid (Spain), our group has recently published the experience of the GMTH in a cohort of 19 patients, with similar results in terms of engraftment and survival to those reported in unmanipulated haplo-HSCT (19). Following this analysis, we aimed to expand the cohort by including all Spanish patients reported to the GETH-TC registry. To the best of our knowledge, this cohort gathers the largest reported experience of haplo-HSCT in patients with DSAs.

Although monitoring and desensitization strategies were mostly personalized, with both intra- and inter-center heterogeneity, some consistent data were found in our study. First, the incidence of engraftment in our cohort was similar to that reported by other groups in patients with DSAs receiving a homogeneous desensitization strategy (18). Our patients were mostly treated based on baseline MFI, with a trend towards a more intensive approach in patients with MFI >5,000. Although other previously described important information including data on complement fixation and post-desensitization intensity was missing in a number of cases, the use of a desensitization approach for patients with intensity >5,000 might have mitigated the detrimental effect of baseline DSA intensity in our cohort, highlighting the importance of this cut-off level. Second, a post-infusion increase in titers was identified as an independent risk factor for GF in our cohort. Although this result should be taken with caution due to the relatively low number of patients monitored and it should be confirmed in other studies, this fact highlights the importance of MFI monitoring after infusion and the need for a continuous desensitization strategy for these patients, as previously reported (10)**;** this should be considered as most centers do not have access to DSA intensity results in a timely manner. Moreover, the previously reported cut-off level for baseline intensity of >20,000 as a risk factor for survival (18) was also confirmed in our cohort. Therefore, in patients with this very high intensity DSA, alternatives including change of donor should be examined. The possibility of using highly MMUD against whom no antibodies are present should be considered in this very high-risk population (26, 27). Finally, regarding complement fixation, in those patients who were studied, nearly 75% showed complement fixation. Moreover, in the three patients with GF and intensity <5,000 MFI, additional factors such as not-detected complement fixation could have been the underlying cause of non-engraftment. Thus, if this technique is not available for patients with MFI <5,000, an assumption of positive fixation might be a safer approach than avoiding desensitization. Our rate of graft failure in this population with <5,000 MFI is higher than that reported by other authors that have reported an absence of impact of these low intensity levels of engraftment (28). However, we should also address there was one case of primary GF in a patient with MFI < 5,000 who received a suboptimal bone marrow graft with a total nucleated count under 2x10^8^/kg, highlighting the importance of a sufficient cell dose, especially in this setting.

Regarding transplant outcomes, although the OS of our cohort was similar to that reported by other groups (18, 19), we found a relatively high NRM rate as compared with the classic rates reported in haplo-HSCT with post-transplant Cy (29), mainly because of infection and endothelial toxicity or bleeding, excluding patients with GF. Factors related to OS were baseline intensity of DSAs > 20,000 MFI and use of a RIC regimen; the second factor might be biased due to the high proportion of patients with prior HSCT, older age and poorer HCT-CI in that group, which may have affected the analysis of OS, EFS and NRM. However, only three patients presented baseline intensity > 20,000 MFI and this result should also be validated in further studies. The impact of DSAs, their intensity and the desensitization strategy on NRM and endothelial toxicity has been studied, but no relationship has been found. Interestingly, the proportion of patients with endothelial toxicity in our cohort appeared to be higher than that reported in haplo-HSCT (30, 31); however, the heterogeneity of the population and other concomitant risk factors such as prior drugs used may affect this result. Multivariate analysis was performed to identify specific risk factors related to DSAs, but we were unable to detect any impact. Despite this high rate of NRM could be circumstantial, we cannot discard a possible effect of the presence of DSAs or their treatment. Thus, it should be considered in patients with a possibility of finding a donor against whom no DSAs are present, especially in those with MFI > 20,000. On the other hand, the incidence of GVHD was lower than expected with a low related mortality. While possible impact of the desensitization strategy could be hypothesized, we could not attribute any effect. Further studies are needed to study the impact of desensitization on GVHD development. Finally, we also found delayed platelet engraftment, with only 35% patients engrafting at day 28 and 64% at day 100; this delayed engraftment in patients with DSAs was also reported in the Madrid cohort and the MDACC experience (18, 19). Whether DSAs have played a role in this delay can be hypothesized but not proven in the setting of haplo-HSCT with post-transplant Cy, in which poor graft function has also been reported (25). Neutrophil engraftment was also delayed in four patients, but may be at least partially explained by concomitant severe complications.

Among the limitations of this study, the design of the survey might have introduced some bias, including selection bias, and it also has all the intrinsic limitations of retrospective studies. Both the population and protocols included were heterogeneous, and the long study period also introduces heterogeneity, as there was a complete lack of guidelines at the beginning of the period. Nevertheless, our study includes a large cohort of patients with a very detailed report of monitoring performed and desensitization strategies used, analysis of possible GF causes, and data on toxic complications, infections and GVHD. All this information validates the risk factors that have previously been described and the usefulness of the recently published guidelines. Our report indicates a need to unify strategies, as previously reported by part of our group in the Madrid experience (19). Analysis of this experience could also guide the creation of local guidelines for the management of these patients. Moreover, the validation of the importance of very high intensity DSAs and their impact on survival underlines the need for alternative strategies in patients with baseline intensity over 20,000 MFI.

In conclusion, the optimal strategy for DSA desensitization is still unclear. Accordingly, the risk factors described in recent guidelines and publications including the importance of DSA intensity, persistence or increase in intensity at infusion and complement fixation should be taken into account in the personalized strategy applied to each patient to make this approach safe for patients who lack an alternative donor.

## Data availability statement

The original contributions presented in the study are included in the article/**Supplementary Material**. Further inquiries can be directed to the corresponding author.

## Ethics statement

The studies involving human participants were reviewed and approved by COMITÉ de ÉTICA DE LA INVESTIGACIÓN con MEDICAMENTOS HOSPITAL GENERAL UNIVERSITARIO GREGORIO MARAÑÓN. Written informed consent for participation was not required for this study in accordance with the national legislation and the institutional requirements.

## Author contributions

Conception and design: RB and MK. Provision of study materials or patients: All authors. Data collection: RB, RA, BH-D, CA-F, AV, AE, MF, LS, IS-V, GB, LB, OL-G, AP-M, AT, JZ and MC. Data analysis and interpretation: All authors. Manuscript writing: RB and MK. Revision and final approval of manuscript: All authors. All authors contributed to the article and approved the submitted version.

## Glossary

**Table:**

Allo-HSCT	Allogeneic hematopoietic stem cell transplantation
BM	Bone marrow
CMV	Cytomegalovirus
DSA	Donor-directed anti-HLA-specific allo-antibody
EFS	Event-free survival
GETH-TC	Spanish Group of Hematopoietic Transplant and Cell Therapy
GF	Graft failure
GMTH	Madrid Group of Hematopoietic Transplant
GVHD	Graft-versus-host disease
Haplo-HSCT	Haploidentical hematopoietic stem cell transplantation
HCT-CI	Hematopoietic stem cell transplant comorbidity index
IVIG	Intravenous immunoglobulins
HLA	Human leukocyte antigen
MFI	Mean fluorescence intensity
MUD	HLA-matched unrelated donor
MMUD	HLA-mismatched unrelated donor
NRM	Non-relapse mortality
OS	Overall survival
PBSC	Peripheral blood stem cells
SOS	sinusoidal obstruction syndrome
TPE	Therapeutic plasma exchange
UCB	Umbilical cord blood.
