# Supplementary material for: Results of haploidentical transplant in patients with donor-specific antibodies: a survey on behalf of the Spanish Group of Hematopoietic Transplant and Cell Therapy
Source: Front Immunol. 2023 May 26;14:1165759. doi: 10.3389/fimmu.2023.1165759 (PMC10250708; doi:10.3389/fimmu.2023.1165759)

**Supplementary Table 1. Causes of death.** Abbreviations: CNS, central nervous system; GVHD, graft-versus-host disease; HSCT, hematopoietic stem cell transplantation; NRM, non-relapse mortality: SOS, sinusoidal obstruction syndrome; TA-TMA, transplant-associated thrombotic microangiopathy.

|  | **Patients**  ***n* = 69** |
| --- | --- |
| 2-year NRM cumulative incidence | 42.6% |
| Total deaths (complete follow up) (n) | 41 |
| Relapse* | 10 (24%) |
| Primary graft failure**   - *Salvage HSCT complication* - *Infection* - *Hemorrhage* | 7 (17%)  *3*  *3*  *1* |
| Acute GVHD-related mortality   - *Refractory/progressive GVHD* - *Infection during treatment* | 6 (15%)  *3*  *3* |
| Infection (not GVHD-related)***   - *Bacterial infection* - *Viral infection* | 9 (22%)  *7*  *2* |
| Endothelial & other toxicity****   - *SOS* - *Hemorrhage* - *TA-TMA* - *Capillary leak syndrome* - *CNS demyelinating disease* | 9 (22%)  *4*  *2*  *1*  *1*  *1* |

*Among relapsed patients, 5 relapsed within the first year after transplant in a median of 5.2 months, and 5 patients relapsed after the first year after a median of 29 months.

**4 out of 7 patients experiencing primary graft failure underwent a 2nd transplant and 1 CD34+ selected boost; 3 out of these 4 patients died due to complications of the salvage transplant including 2 infections and 1 hemorrhage. One patient was alive at last follow up.

***5 patients died during the first 3 months after transplant due to infection, including 2 patients who died in the first month prior to neutrophil engraftment. Regarding the remaining 4 patients, 2 died at 21 and 42 months, respectively, due to late bacterial infections (pneumonia).

****4 patients in this group died prior to day 28 without neutrophil engraftment. All but one patient (CNS complication) in this group died in the first 4 months after HSCT. Among the 2 patients with hemorrhage, 1 died due to CNS bleeding in the context of refractoriness to platelet transfusion and 1 due to pulmonary hemorrhag

**Supplementary Figure 1. Desensitization treatments used.** Abbreviations: DSA, donor specific antibodies; MFI, mean fluorescence intensity; GF: graft failure; IVIG: intravenous immunoglobulin; TPE: therapeutic plasma exchange; IS: immunosuppressors.


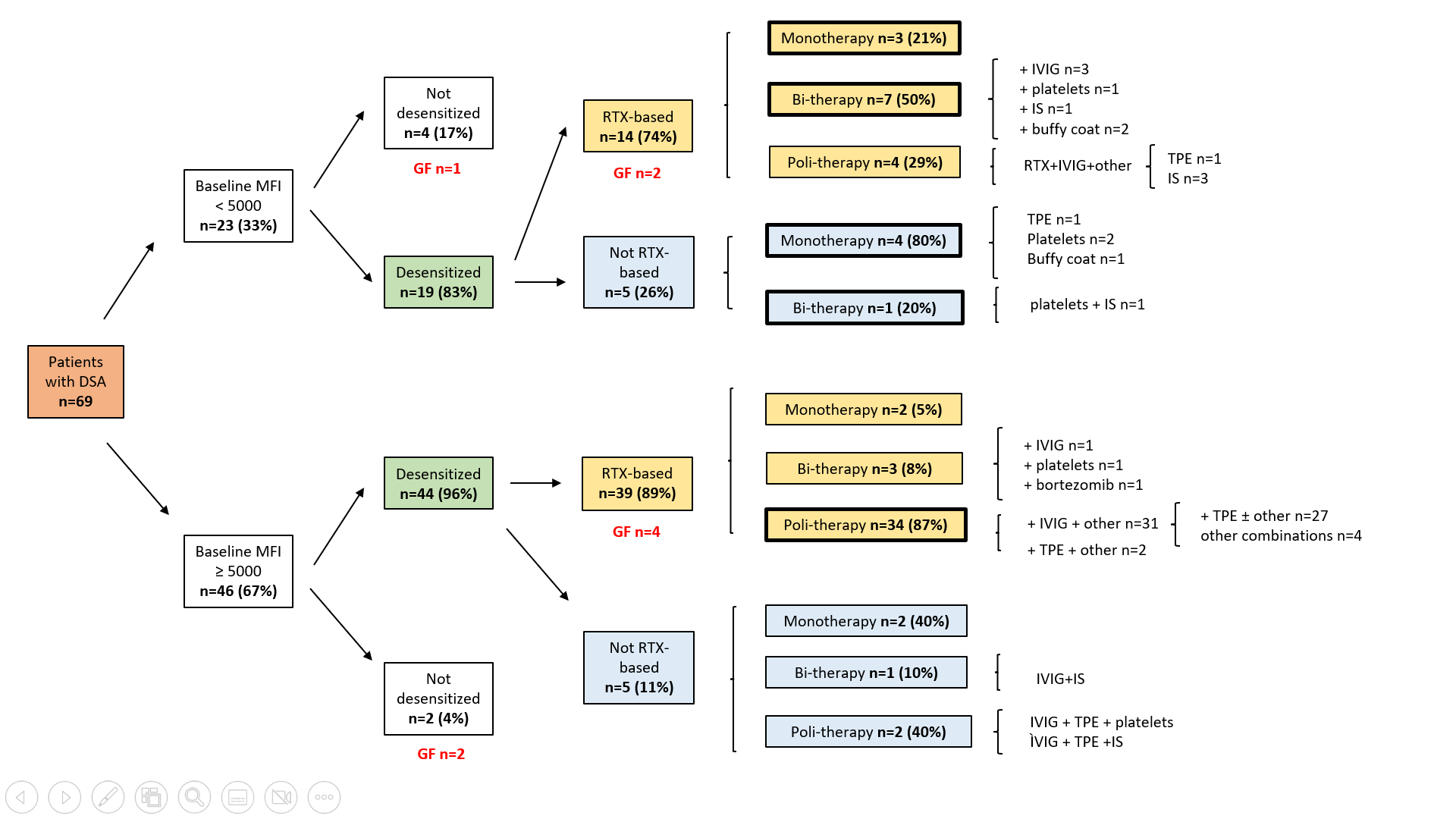


**Supplementary Figure 2. Graft-versus-host disease.** A. Acute GVHD grades II-IV. B. Acute GVHD grades III-IV. C. Chronic GVHD. D. Moderate to severe chronic GVHD.


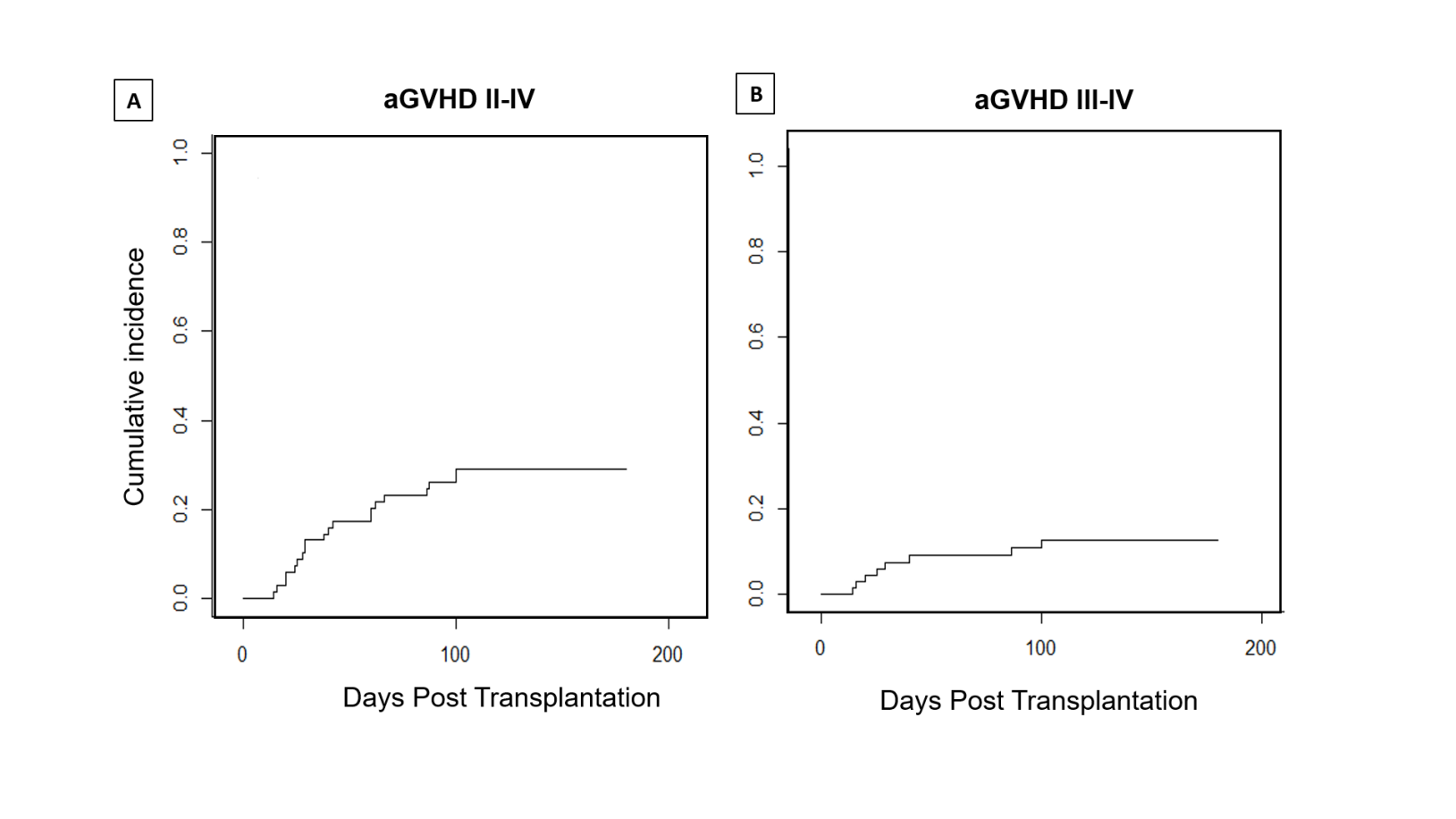


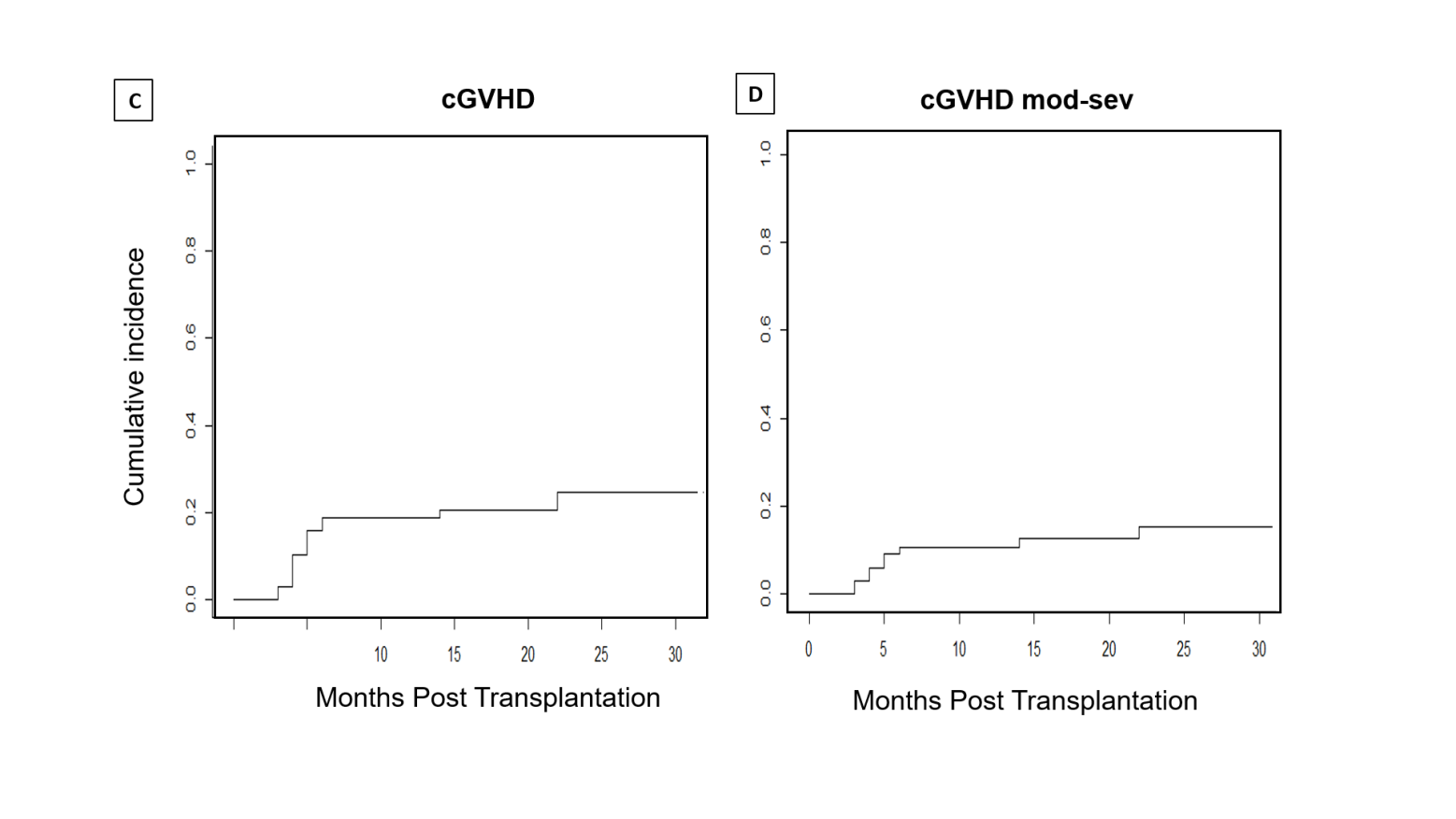

Supplement: Supplementary file 1 [file DataSheet_1.docx]
